# Asymptomatic hepatic artery dissection early after living-donor liver transplantation with simultaneous splenectomy: two case reports

**DOI:** 10.1186/s12876-020-01528-0

**Published:** 2020-11-12

**Authors:** Keita Shimata, Yasuhiko Sugawara, Tomoaki Irie, Yuzuru Sambommatsu, Masashi Kadohisa, Sho Ibuki, Seiichi Kawabata, Kaori Isono, Masaki Honda, Hidekazu Yamamoto, Taizo Hibi

**Affiliations:** Department of Transplantation and Pediatric Surgery, Kumamoto University Hospital, 1-1-1, Honjo, Chuo-ku, Kumamoto, 860-8556 Japan

**Keywords:** Hepatic artery dissection, Living donor liver transplantation, Splenectomy, Intimal dissection, Case report

## Abstract

**Background:**

Hepatic artery dissection after liver transplantation is an uncommon morbidity. The onset mechanism and management for this disorder remain unclear. The present report describes the cases of two patients with hepatic artery dissection after living-donor liver transplantation (LDLT) with simultaneous splenectomy and provides new insight into the onset mechanism of this disorder.

**Case presentation: Case 1:**

A 51-year-old man with liver cirrhosis caused by hepatitis B virus underwent LDLT with a right lobe graft and splenectomy simultaneously. The recipient’s right hepatic artery had partial dissection at the anastomosis site; therefore, his left hepatic artery was anastomosed. Contrast-enhanced computed tomography (CT) on postoperative day (POD) 27 showed dissection from his celiac artery to his left hepatic artery with bleeding in the false lumen. There was a risk of rupture of the false lumen; therefore, emergency interventional radiology and coil embolization of the false lumen were performed. The patient was doing well at 6 months after LDLT.

**Case 2:**

A 58-year-old woman with liver cirrhosis caused by primary biliary cholangitis underwent LDLT with a left lobe graft and splenectomy simultaneously. Her hepatic artery had a dissection that extended from her left hepatic artery to the proper hepatic artery. The gastroduodenal artery was anastomosed. Contrast-enhanced CT on POD 8 revealed dissection from the celiac artery to the common hepatic artery as well as a pseudoaneurysm at the celiac artery. We managed the patient with conservative treatment and performed daily follow-ups with Doppler ultrasonography examination and serial contrast-enhanced CT. At the time of writing this report, the patient was doing well at 34 months after LDLT.

**Conclusions:**

Patients who have an intimal dissection at the anastomosis site and/or simultaneous splenectomy are at a higher risk of hepatic artery dissection. Most patients with asymptomatic hepatic artery dissections can be treated conservatively. Blood flow in the intrahepatic artery should be checked frequently using Doppler ultrasonography or contrast-enhanced CT soon after diagnosis.

## Background

In Asian countries, living donor liver transplantation (LDLT) is widely accepted as a crucial therapeutic option for patients with end-stage liver disease. Patients who undergo LDLT tend to have various vascular complications because of the reconstruction complexity of the vascular structures [1]. Hepatic artery complications that occur after liver transplantation are an uncommon morbidity that may lead to graft loss or the need for emergent re-anastomosis or re-transplant [2–4]. Hepatic artery dissection is a rarely reported hepatic artery complication. The clinical significance, onset mechanism, treatment strategies, and outcomes of hepatic artery dissection remain unclear. Here, we describe the cases of 2 adult patients with hepatic artery dissection after LDLT with simultaneous splenectomy. We describe the characteristic serial computed tomography (CT) images obtained in the month following diagnosis, and provide new insights into the onset mechanism of this complication.

## Case presentation

### Case 1

A 51-year-old male patient developed decompensated liver cirrhosis due to hepatitis B virus and underwent LDLT with a right lobe graft donated by his wife. The ABO-blood-type compatibility between them was identical. The graft-to-recipient weight ratio was 0.78%. He did not have medical history of diabetes mellitus, hypertension and hyperlipidemia. Preoperative CT showed slight arterial calcification on his abdominal aorta.

The hepatic arteries of the recipients were carefully dissected to prevent their thermal and physical damage by avoiding the use of an energy device and pulling a vascular tape. The recipient’s right hepatic artery had partial dissection, and the left hepatic artery was anastomosed with the right hepatic artery of the liver graft in an end-to-end fashion with microsurgery, using 8-0 polypropylene interrupted sutures. We only held the adventitia of the HA and did not pinch the intima during arterial anastomosis. Splenectomy was performed simultaneously due to hypersplenism including thrombocytopenia (blood platelet count < 40,000 /µL). The operation time was 957 min and the blood loss was 15,403 ml. Histopathological examination of hepatic artery showed mild intimal dissection.

His immunosuppressive regimen comprised tacrolimus combined with low-dose steroids as per our usual protocol. The target trough levels of tacrolimus were 10–15 ng/mL in the first 2 weeks, around 10 ng/mL in the next 2 weeks, and 5–10 ng/mL thereafter. Steroids were initiated with an injection of 10 mg/kg of methylprednisolone before graft perfusion during the surgery. He received an intravenous injection of 1 mg/kg of methylprednisolone during POD 1–3, 0.5 mg/kg during POD 4–6, and 0.3 mg/kg on POD 7. Subsequently, they were changed to oral administration of prednisolone. Immediately after the operation, Doppler ultrasonography revealed sufficient blood flow into and out of the intrahepatic artery, portal vein, and hepatic vein. The early postoperative course was uneventful. However, on postoperative day (POD) 2, the levels of fibrinogen/fibrin degradation products and d-dimer were sharply elevated. Contrast -enhanced CT on POD 8 showed no thrombosis in the portal vein; however, there was some thrombosis in the splenic vein. Therefore, heparin sodium was administered at 10,000 units per day to prevent portal vein thrombosis. On POD 13, Doppler ultrasonography showed clear attenuation of the hepatic artery flow velocity. On POD 19, the aspartate transaminase (AST) and alanine aminotransferase (ALT) levels were highly elevated (AST: 58-1514 U/L and ALT: 170-2244 U/L). Contrast-enhanced CT revealed thrombosis in the anterior portal vein and an ischemic injury in segment 8 of the graft. Systemic administration of urokinase and antithrombin-3 was started as a thrombolytic therapy for portal vein thrombosis. The portal vein thrombosis gradually diminished. Intravenous administration of heparin sodium was converted to oral administration of warfarin on POD 23.

**Fig. 1:**
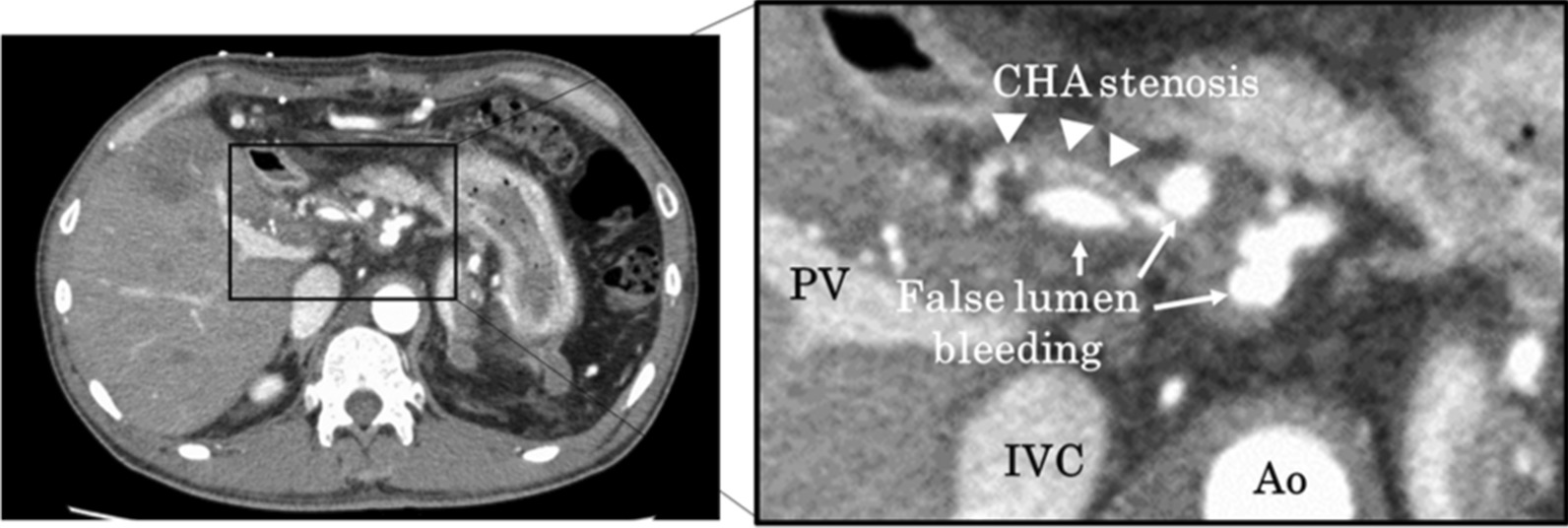
Contrast-enhanced CT in Case 1. The contrast-enhanced CT image showed narrowing of the common hepatic artery (CHA) and some bleeding in the false lumen; the bleeding appeared similar to an aneurysm. Abbreviations: PV, portal vein; IVC, inferior vena cava; Ao, aorta

**Fig. 2:**
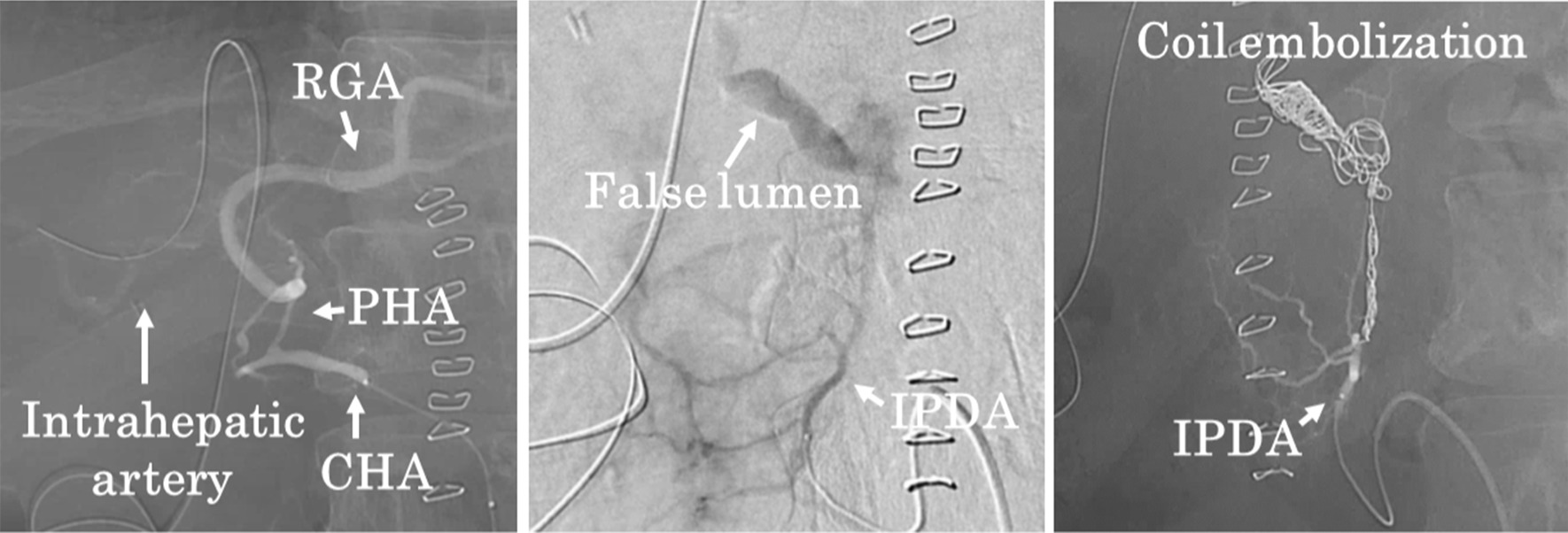
Emergency IVR in Case 1. Angiography with injection from the celiac artery reveals a narrowing proper hepatic artery (PHA), a faint intrahepatic artery, and no false lumens. Angiography with injection from the superior mesenteric artery (SMA) reveals a false lumen around the common hepatic artery (CHA) and a developing collateral circulation that flows to an intrahepatic artery. Coil embolization of the false lumen was performed via the inferior pancreaticoduodenal artery (IPDA). Abbreviations: RGA, right gastric artery

Serial contrast-enhanced CT on POD 27 revealed dissections of the celiac artery, common hepatic artery, proper hepatic artery, and left hepatic artery, as well as bleeding in the false lumen. Contrast-enhanced CT on POD 8 showed a dissection of the hepatic artery without bleeding in the false lumen or aneurysm. Considering the possibility of a rupture of the false lumen or aneurysm, we performed emergent interventional radiology (IVR) that included coil embolization of the false lumen via the inferior pancreaticoduodenal artery (Figs. 1, 2). Doppler ultrasonography examination revealed improved intrahepatic artery flow velocity after IVR. The patient was discharged on POD 44 and was doing well at 10 months following LDLT without recurrence of arterial dissection.

### Case 2

A 58-year-old female patient developed decompensated liver cirrhosis because of primary biliary cholangitis and underwent LDLT with a left lobe graft that was donated by her husband. The ABO-blood-type was compatible. The graft-to-recipient weight ratio was 0.76%. She had no medical history of diabetes mellitus, hypertension, or hyperlipidemia, or arterial calcification on preoperative CT.

Irrespective of careful dissection, a dissection was observed from the recipient’s proper hepatic artery to the left hepatic artery. Thus, the gastroduodenal artery was anastomosed with the left hepatic artery of the liver graft in an end-to-end fashion with microsurgery, using 8-0 polypropylene interrupted sutures. Splenectomy was performed simultaneously due to hypersplenism. The operation time was 824 min, and the blood loss volume was 6051 mL. Histopathological examination of the hepatic artery showed wall thickening without intimal dissection.

Her immunosuppressive regimen comprised tacrolimus combined with low-dose steroids as previously described in Case 1. Immediately after the operation, Doppler ultrasonography showed sufficient blood flow in the intrahepatic artery, portal vein, and hepatic vein. The early postoperative course was uneventful.

**Fig. 3:**
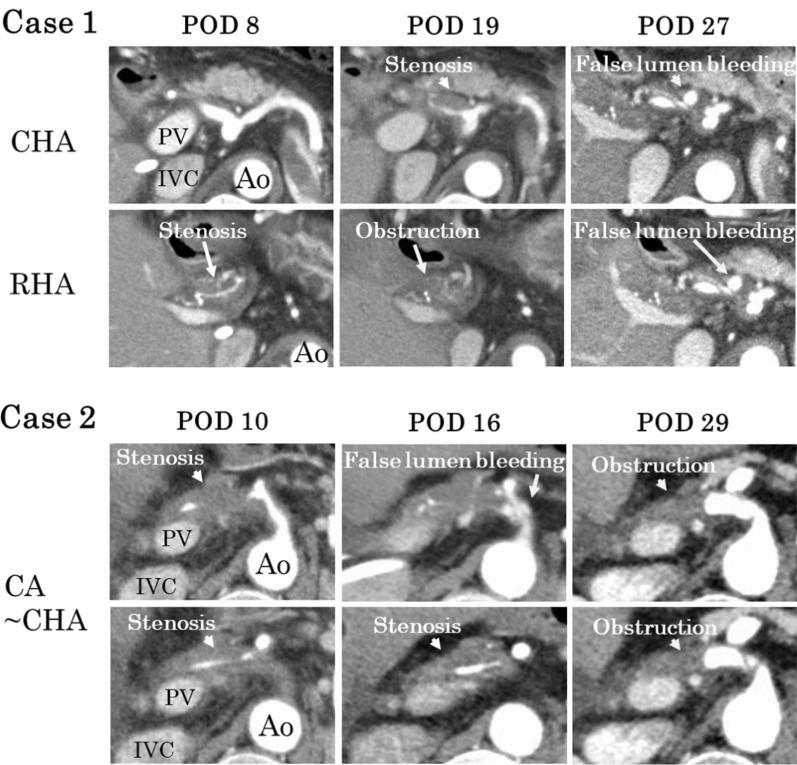
Serial contrast-enhanced CT for Patient 1 and Patient 2. Case 1: Contrast-enhanced CT on postoperative day (POD) 8, 19, and 27 for Patient 1. Contrast-enhanced CT on POD 8 reveals stenosis of the right hepatic artery (RHA) and a low-density area around the common hepatic artery (CHA), suggesting dissection with the formation of a false lumen. There was no bleeding in the false lumen. On POD 19, an obstruction of the RHA and stenosis of the CHA was observed. On POD 27, bleeding in the false lumen was observed around the CHA and the celiac artery (CA). Case 2: Contrast-enhanced CT on POD 10, 16, and 29 for Patient 2. Contrast-enhanced CT on POD 10 reveals a stenosis of the CHA and the CA as well as a low-density area around them. On POD 16, bleeding in the false lumen was observed around the CA. On POD 29, an obstruction of the CHA was observed, and the bleeding in the false lumen around the CHA and the CA increased chronologically. Abbreviations: PV, portal vein; IVC, inferior vena cava; Ao, aorta

On POD 6, Doppler ultrasonography revealed that the hepatic artery flow velocity was attenuated. On POD 7, the ALT and AST levels showed a sharp rise (AST: 56-234 U/L and ALT: 85-354 U/L). Contrast-enhanced CT on POD 8 showed dissection of the common hepatic artery and the celiac artery as well as a pseudoaneurysm at the celiac artery. The Doppler signal of the intrahepatic artery was positive and had a tardus parvus waveform; the AST and ALT levels were decreased, and the patient was treated conservatively and monitored daily with Doppler ultrasonography and serial contrast-enhanced CT examination. Weak intrahepatic artery flow was detected throughout the course. Contrast-enhanced CT on POD 10 and 16 showed dissection of the superior and inferior mesenteric arteries that were observed as perivascular low-density areas. Arteritis was ruled out, based on the results of scintigraphy with ^67^ Ga and serum antibody tests, including antinuclear antibody, myeloperoxidase antineutrophil cytoplasmic antibodies (ANCA), and proteinase 3 ANCA. Oral administration of dipyridamole was started on POD 27 to prevent arterial stenosis. Examination with contrast-enhanced CT on POD 10, 16, and 29 showed chronologic transformation of the stenosis or bleeding in the false lumen (Fig. 3). The patient was discharged to home on POD 47 and was doing well at 62 months following LDLT without recurrence of arterial dissection.

Characteristics and operative data are summarized in Table 1. Date related to HA dissection are summarized in Table 2.

**Table 1:** Characteristics and operative data

	Case 1	Case 2
Age, sex	51 years, male	58 years, female
Primary disease (MELD score)	HBV-LC (17)	PBC (7)
Graft information		
Donor	48 years, wife	67 years, husband
Compatibility	Identical	Compatible
Graft type	Right lobe	Left lobe
GRWR	0.78%	0.76%
Reconstruction	(Donor—Recipient)	(Donor—Recipient)
Hepatic vein	RHV(V5 + V8)—RHV, IRHV—IVC	L + MHV—L + MHV
Portal vein	RPV—PV trunk	LPV—PV trunk
Hepatic artery	RHA—LHA	LHA—GDA
Bile duct	RHD—RHD	LHD—CBD
Existence of intimal dissection	Recipient's RHA	Recipient's LHA and PHA
Splenectomy	Simultaneously	Simultaneously
Operation time	957 min	824 min
Blood loss	15,403 mL	6051 mL

*y* years, *MELD* model for end-stage liver disease, *HBV-LC* hepatitis virus B—liver cirrhosis, *PBC* primary biliary cholangitis, *GRWR* graft-to-recipient body weight ratio, *RHV* right hepatic vein, *IRHV* inferior right hepatic vein, *IVC* inferior vena cava, *L + MHV* left and middle hepatic vein, *RPV* right portal vein, *PV* portal vein, *LPV* left portal vein, *RHA* right hepatic artery, *LHA* left hepatic artery, *GDA* gastroduodenal artery, *RHD* right hepatic duct, *LHD* left hepatic duct, *CBD* common biliary duct

**Table 2:** Data related to HA dissection

	Case 1	Case 2
Diagnosis date	POD 27 (POD 8)	POD 8
Range of dissection	Celiac trunk, CHA, LGA	Celiac trunk, CHA, LGA, SMA, IMA
Symptom	None	None
Diagnosis examination	Contrast-enhanced CT	Contrast-enhanced CT
Weakness of HA flow (date)	Yes (POD 13)	Yes (POD 6)
Flow of intrahepatic artery	Detective	Detective
Comorbidity	Portal thrombosis	Tension headache
	Hypertension	
	Hyperglycemia	
Histopathology of hepatic artery	Mild intimal dissection	Wall thickening, no dissection
Treatment	Coil embolization	Conservative therapy (antiplatelet agent)
Biliary complication	None	None
Follow up period, status	10 months, survival	62 months, survival

*HA* hepatic artery, *POD* postoperative day, *CHA* common hepatic artery, *LGA* left gastric artery, *SMA* superior mesenteric artery, *IMA* inferior mesenteric artery, *CT* computed tomography

## Discussion and conclusion

We report on the cases of 2 patients with early postoperative hepatic artery dissection after LDLT with splenectomy. The dissections were successfully treated with IVR and conservative therapy. The changes in the serial contrast-enhanced CT images of the hepatic artery dissections have also been described. These cases suggest a clinical phenomenon that an intimal dissection at the reconstructed hepatic arteries and simultaneous splenectomy may cause hepatic artery dissection following LDLT. The concept of this complication is new and the onset mechanism requires further investigation.

Hepatic artery dissection after LT derives from an iatrogenic cause at the anastomosis of the hepatic arteries or spontaneous cause at the celiac artery. Clamp injury during the surgery and a honeycomb-like intimal deformity of the recipient’s hepatic artery are possible causes of hepatic artery dissection [5]. Iwaki et al. [6] has reported a case with extensive isolated spontaneous celiac artery dissection after LT. Moreover, Iwaki et al. determined that the dissection of their case spontaneously occurred from the celiac trunk because the entry of the dissection was located on the celiac trunk. However, in the cases of the current study, no entry was noted on the celiac trunk but false lumen on the common hepatic artery in the CT image at the time of onset. The image later showed false lumen bleedings mimicking entry on the celiac trunk (Fig. 3). In addition, hypertension, vasculitis, cystic medial necrosis, connective tissue disorders, and smoking that may cause damage to the arterial intima, are risk factors for isolated spontaneous celiac artery dissection [7, 8]. However, one of the cases of this study had hypertension after surgery, but neither cases of the current study had any risk factors as aforementioned. Therefore, the hepatic artery dissections of the cases of the current study were determined to be initiated from the anastomotic sites of the hepatic arteries and retrogradely extended to the celiac artery.

Intimal dissection at the anastomotic site was already present before the anastomotic procedure in the cases of this study. Intimal dissection of the hepatic artery at the anastomosis site is considered an important risk factor for postoperative hepatic artery complications, including hepatic artery thrombosis and dissection. Lin et al. [9] have reported a reconstructive technique for the hepatic arteries in HCC patients when the intima was dissected during LDLT. In 18 patients, the hepatic artery that was used as the reconstructed artery was dissected at the intima. The artery was trimmed down till the healthy part appeared and then anastomosed with the artery of the liver graft. Two of the patients (11%) had dissected hepatic arteries. One patient had a thrombosis that was treated with re-anastomosis and the other had a dissection that was treated using anti-coagulants. They also reported that no hepatic arterial complications occurred among patients without intimal dissection of the hepatic artery.

Splenectomy may be related to arterial dissection because both our patients underwent splenectomy. Among the 5 patients with hepatic artery dissection that were reported previously [6, 9–11], 2 underwent splenectomy during LT (Table 3). In contrast, during the last decade, only 2 of 51 the patients who underwent LDLT with simultaneous splenectomy in our hospital had undergone hepatic artery dissections. Thus, splenectomy is not an independent risk factor. However, when intraoperative intimal dissection of hepatic artery coexists, simultaneous splenectomy may support the occurrence of hepatic artery dissection via the mechanism shown below. Splenectomy may induce high pressure against the endothelium of the hepatic artery because the large volume of blood that is originally circulating to the spleen refluxes to the liver after splenectomy. The pressure may extend the dissection from the dissected anastomotic site that is dynamically weak. The dissection may also extend retrogradely to the celiac artery, forming a false lumen. Another entry proximal to the initial site and bleeding in the false lumen may be caused by high pressure against the dissected endothelium proximal to the initial entry. Thus, in patients with intimal dissection of the hepatic artery, splenectomy could be a risk factor for hepatic artery dissection following LT.

**Table 3:** Previous and present cases of hepatic artery dissection after LDLT

Study	Sex	Age	Primary disease	Graft type	GRWR	Splenectomy	Intimal dissection at anastomosis	Symptom	Treatment	Outcome
Iwaki et al. [6]	Female	48	Cryptogenic LC	Right lobe	0.85	Yes	None	None	Conservative	18 months, survival
Lin et al. [9]	-	-	-	Right lobe	–	–	Partially	–	Conservative	> 14 months, survival
Kim et al. [10]	Female	54	-	Left lobe	–	–	–	–	–	–
	Male	58	-	Right lobe	–	No	–	–	None	–
Asonuma et al. [11]	Female	30	AIH	Left lobe	0.93	Yes	Partially	None	Re-anastomosis	11 months, death*
Present case 1	Male	51	HBV-LC	Right lobe	0.78	Yes	Partially	None	IVR (coiling)	6 months, survival
Present case 2	Female	58	PBC	Left lobe	0.76	Yes	Partially	None	Conservative	57 months, survival

*GRWR* graft weight to recipient body weight ratio, *LC* liver cirrhosis, *AIH* autoimmune hepatitis, *HBV-LC* hepatitis type B virus-liver cirrhosis, *IVR* interventional radiology, *PBC* primary biliary cholangitis

^*^Death due to bleeding from esophageal ulcer

In the present cases, chronologic changes in the hepatic artery dissection were observed on the serial CT images (Fig. 3). In the early phase, a low-density area was observed around the hepatic artery, suggesting dissection with the formation of a false lumen. In the next phase, the dissection progressed to the celiac artery with bleeding in the false lumen. During the late phase, the bleeding in the false lumen increased, appearing like pseudoaneurysms and the entry of a dissection. Hwang et al. [12] reported on 43 patients with hepatic artery dissection. Among these 43 patients, pseudoaneurysmal dilatations were observed in 11 after an average duration of 9.4 d from the time of detection of the hepatic artery dissection. These cases suggest that the formation of hepatic artery dissection often changes chronologically. As per the changes, the degree of hepatic artery stenosis and intrahepatic arterial flow also changes. Therefore, every time Doppler ultrasonography examination is performed, changes will be observed in the intrahepatic arterial flow. During conservative therapy, it is important to evaluate the presence of the intrahepatic arterial flow, not its velocity.

**Fig. 4:**
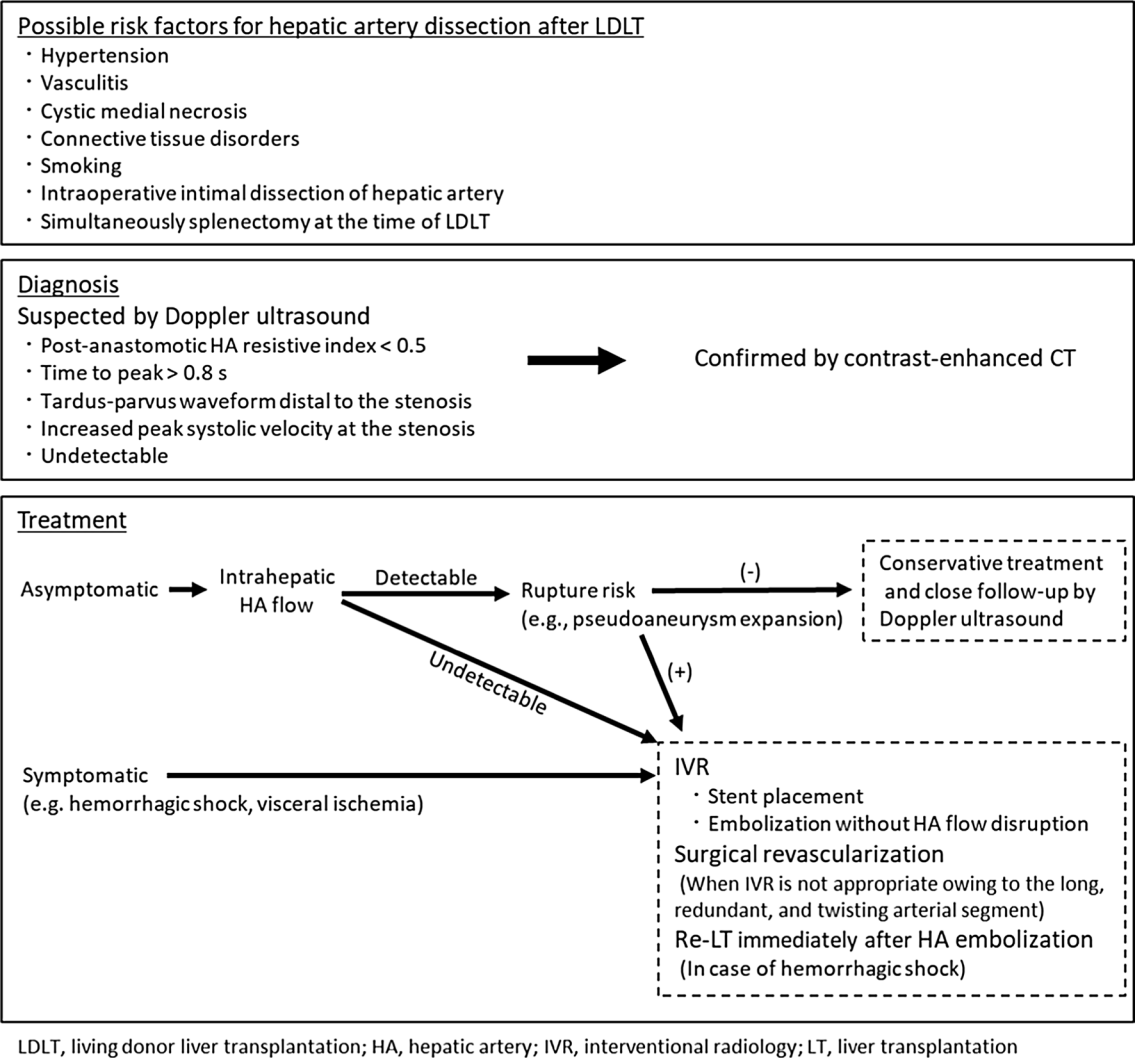
Possible risk factors and strategy of diagnosis and treatment for hepatic artery dissection after LDLT. Abbreviations: LDLT, living donor liver transplantation; HA, hepatic artery; IVR, interventional radiology

Based on previous reports [7, 8, 13, 2] and our experiences of patients who were successfully treated with IVR and conservative treatment, we describe the possible risk factors and strategies for the diagnosis and treatment of hepatic artery dissection following LDLT (Fig. 4). This complication should be suggested on Doppler ultrasonography in the presence of the following features: post-anastomotic HA resistive index < 0.5; time to peak > 0.08 s; tardus-parvus waveform distal to the stenosis; increased peak systolic velocity (> 200 cm/s) at the stenosis; undetectable [13]. If this complication is suspected on Doppler ultrasonography examination, the patient is immediately subjected to enhanced CT for confirming the presence of this complication. Most asymptomatic patients with isolated celiac artery dissection were safely managed with conservative treatment [7, 8]. Furthermore, most patients with hepatic artery dissection after LDLT were resolved with recovery of true lumen without specific treatment [12]. IVR is recommended for patients with dissection progression, aneurysmal degeneration, visceral ischemia, or hemorrhagic shock [8]. For Patient 1, we performed IVR (embolizing the false lumen of common hepatic artery via coiling) because the area of bleeding in the false lumen was enlarged. Therefore, conservative treatment may be selected for patients for whom sufficient intrahepatic artery flow is detected using Doppler ultrasonography or contract-enhanced CT. We detected sufficient flow in the intrahepatic artery and ensured that the hepatic and biliary enzymes were within the normal range during the observation period in Patient 2 who was treated conservatively. The contrast-enhanced CT image on POD 29 showed that the hepatic artery had an adequately sized lumen, and Doppler ultrasonography detected sufficient blood flow in the intrahepatic artery. During conservative treatment, the intrahepatic artery flow should be frequently evaluated using Doppler ultrasonography or contrast-enhanced CT so that treatment for hepatic artery thrombosis can be promptly initiated. In contrast, in symptomatic cases, IVR should be considered to improve the haptic artery flow and prevent the bleeding or rupture from the pseudoaneurysm of the hepatic artery. When IVR is inappropriate owing to the long, redundant, and twisting arterial segment, surgical revascularization can be considered. In case of hemorrhagic shock, re-LT immediately after HA embolization should be considered.

In conclusion, we describe the cases of 2 patients with hepatic artery dissection following LDLT with simultaneous splenectomy. The dissection was initiated at the anastomotic site of the hepatic artery and extended to the celiac artery because of high pressure against the endothelium of the hepatic artery. Asymptomatic hepatic artery dissection can be managed conservatively with frequent evaluation of the intrahepatic artery flow using Doppler ultrasonography or contrast-enhanced CT for few months after establishing the diagnosis of hepatic artery dissection.

## Data Availability

The datasets used or analyzed during the current study are available from the corresponding author on reasonable request.

## Abbreviations

LDLT: Living donor liver transplantation
CT: Computed tomography
POD: Postoperative day
AST: Aspartate transaminase
ALT: Alanine aminotransferase
IVR: Interventional radiology
ANCA: Antineutrophil cytoplasmic antibodies
